# Dopamine Synthesis in the Nigrostriatal Dopaminergic System in Patients at Risk of Developing Parkinson’s Disease at the Prodromal Stage

**DOI:** 10.3390/jcm13030875

**Published:** 2024-02-02

**Authors:** Victor Blokhin, Ekaterina N. Pavlova, Elena A. Katunina, Marina R. Nodel, Galina V. Kataeva, Elina R. Moskalets, Tatiana S. Pronina, Michael V. Ugrumov

**Affiliations:** 1 Laboratory of Neural and Neuroendocrine Regulations, Koltzov Institute of Developmental Biology of Russian Academy of Sciences, Moscow 119334, Russia; victor.blokhin@hotmail.com (V.B.); guchia@gmail.com (E.N.P.); tatiana.pronina@mail.ru (T.S.P.); 2 Federal Center of Brain Research and Neurotechnologies of the Russian Federal Medical and Biological Agency, Moscow 117513, Russia; elkatunina@mail.ru; 3 Faculty of Medicine, Department of Neurology, Neurosurgery and Medical Genetics, N.I. Pirogov Russian National Research Medical University of the Ministry of Health of the Russian Federation, Moscow 117997, Russia; 4 Department of Nervous Diseases and Neurosurgery, Sechenov First Moscow State Medical University (Sechenov University), Moscow 119435, Russia; nodell_m@yahoo.com; 5 Federal State Budget Institution Granov Russian Research Center of Radiology and Surgical Technologies Ministry of Health of the Russian Federation (RRCRST) 70, Leningradskaya Street, Pesochny, St. Petersburg 197758, Russia; kataevagalina@mail.ru; 6 European Medical Center, Moscow 129090, Russia; moskalets.el@gmail.com

**Keywords:** Parkinson’s disease, patients, prodromal, early diagnosis, nigrostriatal dopaminergic system, positron emission tomography, 18F-DOPA

## Abstract

Parkinson’s disease (PD) is diagnosed by the onset of motor symptoms and treated long after its onset. Therefore, the development of the early diagnosis of PD is a priority for neurology. Advanced methodologies for this include (1) searching for patients at risk of developing prodromal PD based on premotor symptoms; (2) searching for changes in the body fluids in these patients as diagnostic biomarkers; (3) verifying the diagnosis of prodromal PD and diagnostic-value biomarkers using positron emission tomography (PET); (4) anticipating the development of motor symptoms. According to our data, the majority of patients (*n* = 14) at risk of developing PD selected in our previous study show pronounced interhemispheric asymmetry in the incorporation of 18F-DOPA into dopamine synthesis in the striatum. This was assessed for the caudate nucleus and putamen separately using the specific binding coefficient, asymmetry index, and putamen/caudate nucleus ratio. Interhemispheric asymmetry in the incorporation of 18F-DOPA into the striatum provides strong evidence for its dopaminergic denervation and the diagnostic value of previously identified blood biomarkers. Of the 17 patients at risk of developing prodromal PD studied using PET, 3 patients developed motor symptoms within a year. Thus, our study shows the promise of using the described methodology for the development of early diagnosis of PD.

## 1. Introduction

Parkinson’s disease (PD) is the second neurodegenerative disease after Alzheimer’s disease in terms of social significance, the incidence and cost of treatment and rehabilitation [1,2]. Most patients suffer from idiopathic or sporadic PD, a multifactorial disease caused by a combination of genetic and epigenetic factors (age, environment, lifestyle, chronic neuroinflammation, traumatic brain injury, etc.) [3,4,5,6,7]. The development of sporadic PD is promoted by toxic factors that affect central and peripheral neurons, including nigrostriatal dopaminergic neurons that control motor function [2]. Endogenous toxins include misfolded aggregated proteins, synuclein and tau [8,9]. The key mechanism of PD pathogenesis is associated with impaired metabolism of α-synuclein, which is normally involved in neurotransmission [10]. It becomes toxic as a result of intraneuronal accumulation due to the disruption of proteasomal degradation by ubiquitin-C-terminal hydrolase L1 and β-glucocerebrosidase [9,11,12]. Tau proteinopathy, like α-synucleinopathy, promotes neurodegeneration, but through impaired axonal transport, which leads to impaired neurotransmission, the degradation of axonal synaptic terminals, and neuronal degeneration [2]. In addition to endogenous neurotoxins, toxic environmental factors play an important role in the pathogenesis of PD. Thus, microbiota toxins entering the body cause α-synucleinopathy with a subsequent prion-like spread of oligomeric α-synuclein from neuron to neuron [7,13,14]. Exogenous neurotoxic factors also include heavy metals, pesticides, etc., that enter the brain intranasally. They accumulate in dopaminergic neurons of the substantia nigra, causing oxidative stress and neurodegeneration [7,15].

Despite the enormous efforts of physicians and researchers, the treatment of PD patients continues to be insufficiently effective [16]. This is explained by the fact that PD is diagnosed according to the developing symptoms of parkinsonism [17] and begins to be treated many years after the onset of the disease. By this time, more than half of the nigrostriatal dopaminergic neurons die, which leads to dopamine deficiency in the striatum, a key link in the regulation of motor function [18,19,20]. Proceeding from this fact, an important trend in current neurology is the development of the early diagnosis of PD long before the emergence of motor symptoms, as well as the development of preventive neuroprotective therapy that slows down the death of neurons. It is assumed that the development of both technologies will make it possible to prolong the early asymptomatic (preclinical) stage of the disease or, in other words, the period of social and physical activity of the patient [2].

Nowadays, PD can be diagnosed 5–10 years before the onset of motor symptoms by identifying the functional insufficiency of the nigrostriatal dopaminergic system with positron emission tomography (PET) using radiolabeled markers of dopaminergic neuron activity [21,22,23,24]. Although various radiolabeled markers can be used for this purpose, 18F-DOPA is considered to be the most informative for assessing dopamine synthesis [25,26]. The main pathological characteristic of the nigrostriatal dopaminergic system in PD, detected using PET and 18F-DOPA, is an interhemispheric asymmetry in dopamine synthesis due to the asymmetric dopaminergic denervation of the striatum (caudate nucleus and putamen) [27,28,29].

Despite the diagnostic attractiveness of PET, this method cannot be used for the preventive examination of the population due to its technical sophistication and high cost. Therefore, at present, the development of an early (preclinical) diagnosis of PD is based primarily on the search for changes in body fluids, mainly in the blood and cerebrospinal fluid in patients in the prodromal (preclinical) stage of PD. PD in the prodromal stage is diagnosed by the manifestation of premotor symptoms characteristic of this disease, which precede the development of motor symptoms [2,28,30,31]. In the future, it will probably be possible to diagnose PD in the prodromal (preclinical) stage using these biomarkers, followed by verification of the diagnosis using PET. PET diagnosis of PD at the prodromal stage, as at the clinical stage, would be based mainly on an asymmetric decrease in the accumulation of 18F-DOPA in the putamen and caudate nucleus in the left and right hemispheres [32].

The purpose of the present article is to evaluate, using 18F-DOPA-PET, the functional state of the nigrostriatal dopaminergic system in terms of dopamine synthesis in patients at risk of developing PD at the prodromal stage, selected and characterized in our previous study, and thus to verify the diagnosis [31].

## 2. Materials and Methods

### 2.1. Patients and Procedures

In this work, we studied the functional state of the nigrostriatal dopaminergic system of the brain using 18F-DOPA-PET in patients at risk of developing Parkinson’s disease (PD) in the prodromal stage, selected in our previous work from a large number of people manifesting premotor symptoms (RBD-REM, smell disorder, constipation, etc.) characteristic of PD at this stage [31]. As shown in previous studies, per a healthy population of 100,000 aged 50 to 54, prodromal PD prevalence is about 0.4%; it is 0.75% from ages 55 to 59; 1.25% from ages 60 to 64; and 2.0% from ages 65 to 69; 2.5% from ages 70 to 74 [33,34]. Our clinical testing identified 26 patients at risk of developing PD in the prodromal stage [31], but only 17 of them agreed to undergo PET examination and signed informed consent.

The main requirements for selecting people at risk of developing PD in the prodromal stage include (i) and age of 55–75 years; (ii) no organic pathology of the central nervous system according to medical history; and (iii) sleep disorder. Each patient was required to score at least 5 points out of 12 points on the REM Sleep Behavior Disorder Screening Questionnaire (RBDSQ) [35].

Secondary requirements for risk-selected patients include (i) olfactory impairment identified using the Sniffin’Sticks test system (Burghart Medizintechnik, Wedel, Germany), confirmed via at least 12 incorrect odor identifications out of 16 possible ones; (ii) complaints of impaired intestinal motility, confirmed via a positive answer to 2 of 3 questions on the SCOPA-AUT scale (Outcome Scale for Parkinson’s Disease—Autonomic Dysfunction); (iii) psychoemotional disorders documented by a score of at least 8 out of 21 possible on the depression or anxiety subscale of the Hospital Anxiety and Depression Scale [36]; (iv) mild movement disorder, confirmed by receiving a score of more than 2 but not more than 6 points out of 108 possible points on the motor symptom assessment of Part III (Movement Disorder) of the Unified Parkinson’s Disease Rating Scale (UPDRS) [37], that meets the criteria for the MDS Prodromal Parkinson’s Disease Study [33,38]. Patients who met all primary requirements and at least one secondary requirement were included in the trial. The criteria for the inclusion of patients in the trial are described in more detail in our previous article [31]. Patients included in the risk group continued to be under neurological observation for a year after the PET study.

The PET study was conducted in accordance with the Declaration of Helsinki and approved by the Institutional Ethics Committee of the Koltzov Institute of Developmental Biology of the Russian Academy of Sciences (protocol No. 55 and date of approval 9 December 2021). The study was performed in accordance with the ethical standards described in the Declaration of Helsinki (https://www.wma.net/policies-post/wma-declaration-of-helsinki-ethical-principles-for-medical-research-involving-human, (accessed on 5 October 2023)). Written informed consent was obtained from all participants.

The PET/CT was performed at the same time of the day, from 2 pm to 5 pm at the European Medical Center (EMC, Moscow, Russia), guided by previously published recommendations for the use of 18F-DOPA [39]. The patients, regardless of body weight, were injected with 250 ± 15 MBq (mean ± standard deviation) of 18F-DOPA into the median cubital vein, using an automatic injector, Intego 2010 (MEDRAD Inc., Warrendale, PA, USA). Briefly, 18F-DOPA was manufactured on a Cyclone 18/9 cyclotron (IBA RadioPharma Solutions, Louvain-La-Neuve, Belgium), which included two automated modules for “Synthera” synthesis with computer remote control (IBA, Louvain-La-Neuve, Belgium). In accordance with the methodological recommendations of EANM for dopaminergic imaging in Parkinsonian syndromes, the patients abstained from eating for 4–6 h prior to the administration of 18F-DOPA [39]. The PET/CT examination was performed using the following CT scanners: Biograph mCT40 (Siemens, Berlin, Germany) and Gemini TF TOF 64T (Philips, Amsterdam, The Netherlands). Scanning began 90 min after 18F-DOPA administration and lasted for 20 min. PET/CT was performed in the helical mode without contrast enhancement at an X-ray tube voltage of 100 kV and a current of 100 mA*s in the Care Dose mode and with a virtual slice thickness of 2 mm.

### 2.2. PET Data Analysis

Regions of interest (ROIs) for the analysis, putamen and caudate nucleus in the left and right hemisphere, as well as the left occipital cortex (Locc) and right occipital cortex (Rocc) as a reference zone, were delineated manually, and mean ROI uptake, normalized to body weight and injected activity, the standardized uptake value (SUV), were calculated with SyngoVia software VB30A_HF08 (Siemens, Berlin, Germany).

For each structure, the following parameters were calculated:

1. Specific binding ratio (SBR_ROI_):
  SBR_ROI_ = mean SUV_ROI_/(meanSUV Locc + meanSUV Rocc) × 0.5
2. Asymmetry index (AI):
  AI = (SBR_in left ROI_ − SBR_in right ROI_)/(SBR_in left ROI_ + SBR_in right ROI_) × 100%.
3. AI absolute value:
  AI = |(SBR_in left ROI_ − SBR_in right ROI_)/(SBR_in left ROI_ + SBR_in right ROI_) × 100%)|.
4. Putamen/Caudate nucleus ratio (P/C ratio):
  P/C ratio = SBR_putamen_/SBR_Caudate_.

### 2.3. Obtaining Color Images in the Lightbox Format

To obtain images after PET/CT scanning, data in DICOM format were loaded into the Slicer 3D v5.2.2 software with additional modules Brain Volume Refinement, DCMQI, PET-IndiC, Quantitative Reporting, and PETDICOMExtension. For the obtained images, the “PET-Rainbow2” color scheme was used in the colors module. In the Utilities section, the images were then saved in the axial plane in the “Lightbox image” format, using the Screen Capture function. Thus, 12 images of the striatum (caudate nucleus and putamen) were obtained for each patient.

### 2.4. Statistics

Statistical data processing was performed using the GraphPad Prism v.9.5.1 software (GraphPad Software, Boston, MA, USA). The paired non-parametric Wilcoxon test was used to process the data. A change was considered significant at *p* < 0.05. Correlation analysis between the minimum SBR value in the brain and the AI for the caudate nucleus and putamen was performed using the non-parametric Spearman test.

## 3. Results

Data for all the calculated indicators are shown in Table 1.

**Figure 1:**
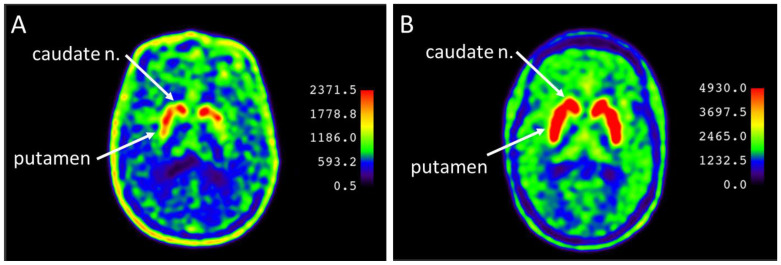
Accumulation of 18F-DOPA in the caudate nucleus (n.) and putamen in patients at risk of developing Parkinson’s disease at the prodromal stage: (**A**) pronounced interhemispheric asymmetry of 18F-DOPA accumulation in the putamen in patient No. 16; (**B**) absence of interhemispheric asymmetry in the accumulation of 18F-DOPA in the striatum in patient No. 14.

Based on the results of PET combined with computed tomography (PET/CT), images were obtained for all patients characterizing the accumulation of 18F-DOPA in the caudate nucleus and putamen (Figure 1).

**Figure 2:**
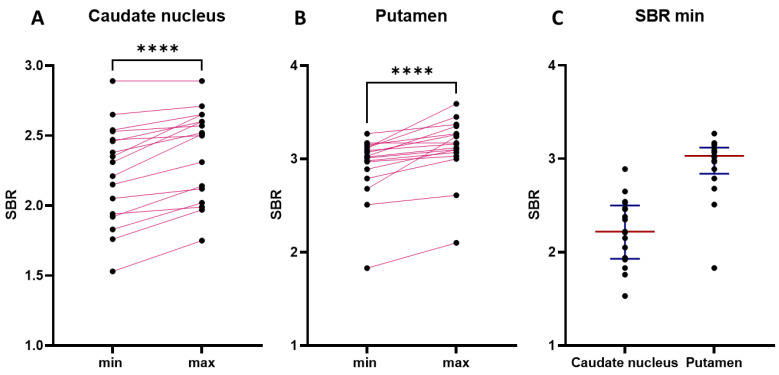
Specific binding ratio (SBR) for the caudate nucleus and putamen in each of the 17 patients at risk of developing Parkinson’s disease at the prodromal stage. (**A**) Minimal and maximal SBR values in caudate nucleus. (**B**) Minimal and maximal SBR values in putamen. (**C**) SBR min variability in the caudate nucleus and putamen. Black dot: specific binding ratio for an individual patient; min: the smallest value for the caudate nucleus and putamen; max: the highest value for the caudate nucleus and putamen; red line: median; blue line: interquartile ranges; pink line connects the values of an individual patient. **** *p* < 0.001.

Quantitative analysis of the specific binding ratio (SBR) in the entire risk group revealed statistically significant interhemispheric differences in the accumulation of 18F-DOPA in the caudate nucleus and putamen (Figure 2). The minimum SBR values in the caudate nucleus ranged from 1.53 to 2.89, while the maximum values ranged from 1.75 to 2.89 (Figure 2A,C).

In the putamen, the minimum SBR values ranged from 1.83 to 3.15, whereas the maximum values ranged from 2.10 to 3.59 (Figure 2B,C).

**Figure 3:**
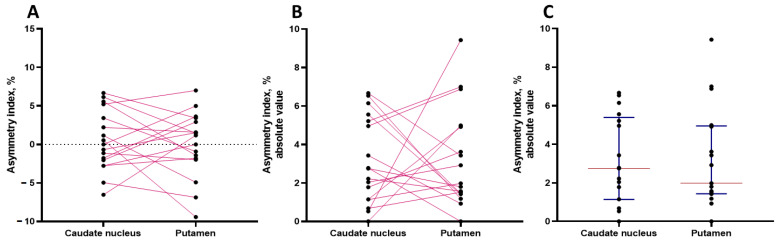
Index of interhemispheric asymmetry in the accumulation of 18F-DOPA in the caudate nucleus and putamen in each of the 17 patients at risk of developing Parkinson’s disease in the prodromal stage. (**A**) Asymmetry index for caudate nucleus and putamen showing lateralization. (**B**) Absolute value of asymmetry. (**C**) Variability of the asymmetry index in the caudate nucleus and putamen. Black dot: asymmetry index for an individual patient; red line: median; blue line: interquartile ranges; pink line connects the values of an individual patient.

The asymmetry index (AI) in patients at risk of developing PD in the prodromal stage showed that in the caudate nucleus, it varies from 0 to 6.67%, and in the putamen, it varies from 0 to 9.43%, in a wider range (Figure 3).

**Figure 4:**
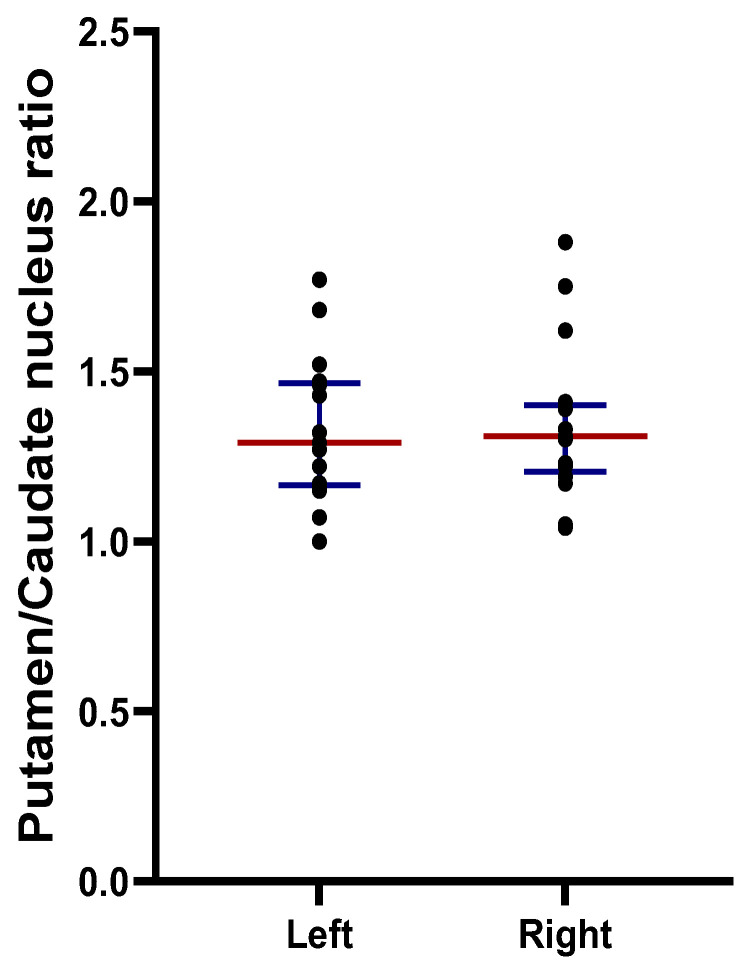
Putamen/caudate nucleus ratio in patients at risk of developing Parkinson’s disease at the prodromal stage. Left: left hemisphere; right: right hemisphere; black dot: ratio for an individual patient; red line: median; blue line: interquartile ranges.

The ratio of the specific accumulation of 18F-DOPA in the putamen to the accumulation in the caudate nucleus (P/C ratio) for the left hemisphere varied from 1 to 1.68, and for the right hemisphere it varied from 1.04 to 1.8 (Figure 4).

**Figure 5:**
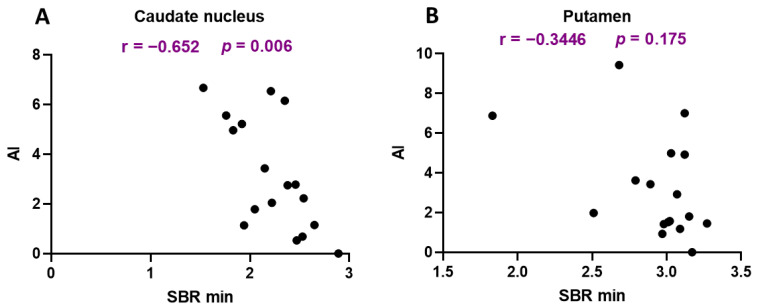
Correlation analysis between the minimum specific binding ratio (SBR min) value and the asymmetry index for the caudate nucleus (**A**) and putamen (**B**). r: Pearson correlation coefficient; *p*: level of significance.

Our correlation analysis between the minimum SBR value and the asymmetry index in the caudate nucleus and putamen revealed an inverse correlation only for the caudate nucleus (Figure 5).

According to neurological examination, three patients at risk of developing PD at the prodromal stage developed motor symptoms characteristic of Parkinson’s disease during the first year after the PET study. Thus, in the first patient (patient No. 16), after 6 months, the following symptoms appeared in the right side: mild postural tremors, hypokinesia when performing motor tests, and rigidity. In addition, the same patient exhibited acheirokinesis. The patient was prescribed therapy as a combination of pramipexole and rasagiline. Until now, two years after the PET study, against the background of treatment, the symptoms are still one-sided. These data indicate the development of PD at the first stage according to the Hoehn–Yahr scale.

In the second patient (patient No. 13), the first motor impairments appeared 8 months after the PET study. Neurological symptoms in this patient are presented in the form of mild hypokinesia and rigidity in the left arm and leg. Over 14 months of neurological observation and treatment with rasagiline, the symptoms remained the same in severity (stage 1, Hoehn–Yahr scale).

In the third patient (Patient No. 4), by the end of the first year, the following mild symptoms appeared in the arm on the right side: postural tremor, terminal tremor, and hypokinesia (stage 1, Hoehn–Yahr scale).

## 4. Discussion

Proceeding from the fact that the relatively low effectiveness of therapy for PD patients is due to the late diagnosis and late onset of their treatment, the most advanced trend in neurology is the development of early (preclinical) diagnosis based on a search for clinical premotor symptoms and changes in the body fluids [2,40,41,42,43]. Such developments include several sequential stages: (i) a search for patients at risk of developing PD at the prodromal (preclinical) stage based on the manifestation of premotor symptoms (RBD, olfactory dysfunction, constipation, etc. [44,45,46]; (ii) a search for changes (biomarkers) in the body fluids of patients at risk of developing PD at the prodromal stage; (iii) a verification of the clinical diagnosis of PD at the prodromal stage and biomarkers in body fluids in selected patients using PET; and finally (iv) the anticipation of the onset of motor symptoms characteristic of PD over time in individual patients at risk of developing PD [47].

This study corresponds to the third and fourth stages of the methodology described above. We used PET to assess the functional state of the nigrostriatal dopaminergic system in terms of dopamine synthesis and validated the biomarkers found in our previous study in the blood of patients at risk of developing PD at the prodromal stage [31]. For PET studies of the nigrostriatal dopaminergic system, various radiolabeled markers can be used. These include (i) markers of the dopamine transporter; (ii) markers of vesicular monoamine transporter 2; (iii) markers of postsynaptic dopamine D2 receptors; and (iv) radiolabeled DOPA, the immediate precursor of dopamine synthesis [22,26,48]. Considering the advantages and disadvantages of each of these radiolabeled markers, 18F-DOPA was selected for use in this study. This marker, which serves to assess dopamine synthesis, is used in most PET studies of patients at the prodromal and clinical stages of PD [26,27,49].

Based on the above, we assessed the functional deficiency of the nigrostriatal dopaminergic system in terms of dopamine synthesis in patients at risk of developing PD at the prodromal stage in accordance with the same criteria that are used in PET studies of PD patients. These include the SBR, AI, and putamen/caudate nucleus ratio. The most informative indicator of the dopaminergic denervation of the striatum in PD patients is interhemispheric asymmetry in the accumulation of 18F-DOPA in this particular brain region [50,51]. In healthy people, the AI is significantly lower than that in patients with PD, being on average 0.95% for the caudate nucleus and 2% for the putamen [52]. According to our data, the AI in the caudate nucleus in 11 out of 17 patients with the risk of developing PD at the prodromal stage was higher than that in in healthy people, based on the research literature data. At the same time, the AI for the putamen was higher in 7 of 17 patients at risk of developing PD. In five of seven patients at risk of developing PD at the prodromal stage, the AI was even higher than that in patients with PD [51]. One of these at risk patients subsequently developed motor symptoms (see below). Moreover, only 4 patients out of 17 in the risk group were characterized by asymmetry in both the caudate nucleus and the putamen. The above data show that some patients selected in our previous study at risk for developing PD began to develop motor symptoms over time, indicating the on-set of the clinical stage of PD.

It should be noted that, along with patients with a pronounced interhemispheric asymmetry of the dopaminergic denervation of the striatum and dopamine synthesis, who have a high AI, we identified patients with virtually no asymmetry. However, one of these patients developed motor symptoms over time. Our data regarding patients at risk of developing PD with high and low AI are consistent with the ideas of Braak et al. [53] and Knudsen et al. [28], who consider that neurodegeneration in PD can begin in the olfactory bulbs of the brain, spread to the periphery, and, vice versa, begin in the intestine and spread towards the brain. It is believed that, despite the dopaminergic denervation of the striatum in both cases, this process is characterized by interhemispheric asymmetry only in the first case.

In addition to the AI, an important semi-quantitative indicator of the asymmetry of 18F-DOPA accumulation in the striatum and therefore the dopamine synthesis and dopaminergic denervation of the striatum in PD is the SBR. In our study, in patients at risk of developing PD, the median minimum value of the SBR in the caudate nucleus was 2.22, which is lower than the median SBR in healthy people, which ranges from 2.4 [54] to 2.9 [27]. From a comparison of our data with the literature data, it follows that in patients at risk of developing PD at the prodromal stage, there is a dopaminergic denervation of the striatum. This correlates well with decreased striatal SBR in PD patients compared with that in healthy controls [27,55]. It is important to note that in our study, 7 out of 17 patients in the risk group had a lower total SBR value compared with the median for the caudate nucleus. From a comparison of these data with changes in the SBR in patients with PD [27,55], it follows that at the prodromal stage, the caudate nucleus undergoes dopaminergic denervation.

Changes in the SBR are also an indicator of the dopaminergic denervation of the putamen [56,57]. In our study, in 17 patients at risk of developing PD in the prodromal stage, the minimum SBR value for the putamen varied from 1.83 to 3.27, and the median was 3.02, which is also typical for healthy people [54,55]. However, among the general population of patients at risk of developing PD, in four patients, the SBR values (1.83, 2.51, 2.68, and 2.79) were lower than the median SBR in patients at risk in general and that in healthy people, which according to Abramov et al. [54] and Oehme et al. [55] are 2.8 and 2.97, respectively. According to Oehme et al. [55], the median SBR value in the putamen in patients with diagnosed PD is significantly lower than that in healthy people, being 2.18. Thus, only 1 of the 17 patients in our group at risk of developing prodromal PD had a lower SBR for the putamen than patients with diagnosed PD [55], indicating the dopaminergic denervation of the putamen. This suggestion, based on the PET examination, was confirmed by the development of motor symptoms over time in this patient (patient No. 16, SBR min = 1.83).

From a comparison of SBRs for the caudate nucleus and putamen for patients at risk of developing PD, it follows that the dopaminergic denervation of the caudate nucleus is more pronounced than the denervation of the putamen. These data are consistent with Pasquini et al.’s research, who reported the dominance of the dopaminergic denervation of the caudate nucleus in the early stage of PD [58].

The third indicator of dopaminergic denervation of the striatum in PD is the ratio of the SBR in the putamen to the SBR of the caudate nucleus (P/C ratio). Most studies have shown that the P/C ratio varies from 1.1 to 1.2 in healthy people, and in PD, the P/C ratio becomes less than one, indicating the preferential denervation of the putamen compared with that of the caudate nucleus. In our group of patients at risk of developing PD, the median P/C ratios in the left and right hemispheres were 1.22 and 1.30, respectively. Individual P/C ratios ranged from 1 to 1.88. Such a high P/C ratio indicates that in the group of patients at risk of developing PD, the caudate nucleus suffers to a greater extent than the putamen. The most convincing evidence for this assumption was that two out of three patients in our group at risk of developing PD with a P/C ratio of 1.41 and 1.88, and therefore with the predominant dopaminergic denervation of the caudate nucleus, developed motor symptoms.

Of particular value are our data on the appearance of motor symptoms in some selected patients at risk of developing PD during the first year after the PET study. In-deed, according to a neurological examination, 3 out of 17 patients at risk of developing PD at the prodromal stage developed motor symptoms, characteristic of PD, within approximately one year after the PET examination. In the first patient, PET revealed pronounced interhemispheric asymmetry in the caudate nucleus (AI = 4.96) and in the putamen (AI = 6.88), while the SBR in both structures was the same: 1.83. Six months after the PET examination, this patient showed postural tremors, hypokinesia when performing motor tests, increased muscle tone of the extrapyramidal type, as well as acheirokinesis when walking. This patient, treated with pramipexole and rasagiline, continued to have unilateral motor symptoms, which corresponds to stage 1 on the Hoehn–Yahr scale.

In the second patient from the risk group for developing PD in the prodromal stage, who developed motor symptoms over time, our PET study showed interhemispheric asymmetry in the caudate nucleus (AI = 6.67) and putamen (AI = 3.44). The minimum SBR was 1.53 in the caudate nucleus and 2.89 in the putamen. Thus, in this patient, although there was no reduction in 18F-DOPA accumulation in the putamen, a pronounced asymmetry in both the putamen and the caudate nucleus was visible. The first left-sided motor symptoms appeared 8 months after the PET examination. These were manifested as mild hypokinesia and increased muscle tone of the extrapyramidal type in the left arm. Over the next 14 months of neurological observation and the treatment of the patient with rasagiline, the severity of the motor symptoms did not change.

In the third patient from the risk group for developing PD at the prodromal stage, our PET study revealed asymmetry only in the caudate nucleus (AI = 3.44), whereas the putamen AI (0.93) did not exceed this indicator for healthy people [52]. In this case, the minimum SBR was 2.15 in the caudate nucleus and 2.97 in the putamen. It is important to note that in this patient, the uptake of 18F-DOPA in the caudate nucleus differed little from that in healthy people, based on the published literature. The P/C ratio was 1.41, which corresponds to the upper limit of the norm. This patient, by the end of the first year after the PET examination, developed mild forms of postural tremor, terminal tremor, and hypokinesia in the right arm.

We continue neurological follow-up in 14 additional patients, believing that some, but not all, will develop motor symptoms over time. Indeed, premotor symptoms used to diagnose prodromal PD are not specific for this disease, and some patients could be mistakenly included in a risk group.

## 5. Conclusions

This study shows that the vast majority of patients at risk of developing PD selected in our previous study [1] exhibit a pronounced asymmetry in the incorporation of 18F-DOPA into the striatum (caudate nucleus and putamen). This is convincing evidence of the dopaminergic denervation of the striatum, a key structure for the regulation of motor function. In addition, the PET study has confirmed the reliability of previously described blood biomarkers [1]. Of the 17 patients at risk of developing prodromal PD studied using PET, three patients developed motor symptoms within a year after the PET examination, which is the most convincing evidence for the correct diagnosis of PD in the prodromal stage. Thus, our study has shown the high potential of using the described technique for the development of the early (preclinical) diagnosis of PD.

Our study and previous similar studies showed the promise of the approach used for the development of preclinical diagnosis of PD. However, given that each individual trial may involve a small number of patients, and that the trial itself is expensive and time-consuming, it would be advisable to conduct multicenter trials using the same protocol to examine patients. In addition, it is advisable to use SPECT-DaTSCAN instead of 18F-DOPA PET. Although both methods have the same high sensitivity and specificity [25,59], SPECT-DaTSCAN is a less expensive and technically more available method [60]. The development of diagnostics of PD at the prodromal stage will allow us to move to the development of preventive neuroprotective treatment. It is assumed that the use of neuroprotectors will slow down neurodegeneration, thereby prolonging the preclinical stage of the patient’s comfortable life.

## Figures and Tables

**Table 1:** Indicators of the functional state of the nigrostriatal system in patients at risk of developing Parkinson’s disease at the prodromal stage.

Patient	Age	Sex	SBR				Asymmetry Index, %		P/C Ratio		OT	RBDSQ	UPDRS
			Caudate Nucleus		Putamen		Caudate Nucleus	Putamen	L	R			
			L	R	L	R							
1	68	M	1.97	1.76	2.98	3.07	5.56	−1.42	1.52	1.75	10	9	2
2	64	M	2.22	2.31	3.26	3.07	−2.04	2.92	1.47	1.33	7	7	0
3	73	F	2.46	2.60	3.17	3.17	−2.78	0.00	1.29	1.22	14	9	5
**4**	75	F	**2.31**	**2.15**	**2.97**	**3.03**	**3.43**	**−0.93**	**1.29**	**1.41**	5	4	3
5	50	F	2.21	2.51	3.16	3.09	−6.54	1.18	1.43	1.23	13	5	1
6	64	M	2.05	2.12	3.00	2.79	−1.78	3.62	1.46	1.31	7	11	0
7	49	M	2.89	2.89	3.35	3.03	0.00	4.99	1.16	1.05	14	0	3.5
8	55	M	2.71	2.65	3.12	3.45	1.15	−4.92	1.15	1.30	8	9	2
9	34	M	2.38	2.52	3.15	3.27	−2.75	−1.80	1.32	1.30	3	5	6
10	55	F	2.65	2.35	3.37	3.27	6.15	1.45	1.27	1.39	10	0	0
11	66	M	2.65	2.54	3.12	3.02	2.22	1.57	1.17	1.19	11	7	0
12	67	M	2.50	2.47	2.68	3.24	0.53	−9.43	1.07	1.31	13	10	0
**13**	71	M	**1.75**	**1.53**	**3.10**	**2.89**	**6.67**	**3.43**	**1.77**	**1.88**	9	10	1
14	48	F	2.53	2.57	3.10	3.01	−0.68	1.52	1.22	1.17	14	5	1
15	57	F	1.94	1.99	2.51	2.61	−1.14	−1.98	1.29	1.31	12	6	0
**16**	58	M	**1.83**	**2.02**	**1.83**	**2.10**	**−4.96**	**−6.88**	**1.00**	**1.04**	14	0	3.5
17	69	M	2.14	1.92	3.59	3.12	5.22	7.00	1.68	1.62	15	4	3

Red bold: patients who developed motor symptoms after 18F-DOPA-PET procedure. SBR—specific binding ratio; P/C ratio—putamen/caudate ratio; L—left; R—right; OT—olfactory test; RBDSQ-REM Sleep Behavior Disorder Screening Questionnaire; UPDRS—unified Parkinson’s disease rating scale.

## Data Availability

Data are available upon request.
